# Estrogen receptor profiles across tissues from male and female *Rattus norvegicus*

**DOI:** 10.1186/s13293-019-0219-9

**Published:** 2019-01-11

**Authors:** Dillion D. Hutson, Rakesh Gurrala, Benard O. Ogola, Margaret A. Zimmerman, Ricardo Mostany, Ryousuke Satou, Sarah H. Lindsey

**Affiliations:** 1 Department of Pharmacology, Tulane University School of Medicine, New Orleans, LA 70112 USA; 2 Tulane Brain Institute, Tulane University School of Medicine, New Orleans, LA 70112 USA; 3 Department of Physiology, Tulane University School of Medicine, New Orleans, LA 70112 USA; 4 Hypertension and Renal Center of Excellence, Tulane University School of Medicine, New Orleans, LA 70112 USA

**Keywords:** Estrogen receptors, Aromatase, Droplet digital PCR (ddPCR), Sex differences, Cardiovascular

## Abstract

**Background:**

Estrogen is formed by the enzyme aromatase (CYP19A1) and signals via three identified receptors ERα (ESR1), ERß (ESR2), and the G protein-coupled estrogen receptor (GPER). Understanding the relative contribution of each receptor to estrogenic signaling may elucidate the disparate effects of this sex hormone across tissues, and recent developments in PCR technology allow absolute quantification and direct comparison of multiple targets. We hypothesized that this approach would reveal tissue- and sex-specific differences in estrogen receptor mRNA.

**Methods:**

ESR1, ESR2, GPER, and CYP19A1 were measured in four cardiovascular tissues (heart, aorta, kidney, and adrenal gland), three brain areas (somatosensory cortex, hippocampus, and prefrontal cortex), and reproductive tissues (ovaries, mammary gland, uterus, testes) from six male and six female adult Sprague-Dawley rats.

**Results:**

GPER mRNA expression was relatively stable across all tissues in both sexes, ranging from 5.49 to 113 copies/ng RNA, a 21-fold difference. In contrast, ESR1/ESR2 were variable across tissues although similar within an organ system. ESR1 ranged from 4.46 to 614 copies/ng RNA (138-fold difference) while ESR2 ranged from 0.154 to 83.1 copies/ng RNA (540-fold). Significant sex differences were broadly absent except for renal ESR1 (female 206 vs. male 614 copies/ng RNA, *P* < 0.0001) and GPER (62.0 vs. 30.2 copies/ng RNA, *P* < 0.05) as well as gonadal GPER (5.49 vs. 47.5 copies/ng RNA, *P* < 0.01), ESR2 (83.1 vs. 0.299 copies/ng RNA, *P* < 0.01), and CYP19A1 (322 vs. 7.18 copies/ng RNA, *P* < 0.01). Cardiovascular tissues showed a predominance of ESR1, followed by GPER. In contrast, GPER was the predominant transcript in the brain with similarly low levels of ESR1 and ESR2. CYP19A1 was detected at very low levels except for reproductive tissues and the hippocampus.

**Conclusion:**

While the data indicates a lack of sex differences in most tissues, significant differences were found in the range of receptor gene expression across tissues as well as in the receptor profile between organ systems. The data provide a guide for future studies by establishing estrogen receptor expression across multiple tissues using absolute PCR quantification. This knowledge on tissue-specific estrogen receptor profiles will aid the development of hormonal therapies that elicit beneficial effects in specific tissues.

## Background

The nuanced relationship that exists between normal physiology, endogenous estrogens, and menopausal hormone therapy (MHT) is highlighted by discrepancies in both basic and clinical research. The alarming publication of deleterious estrogenic effects associated with MHT in the large randomized clinical trial the *Women’s Health Initiative* conflict with numerous basic studies performed in animals as well as clinical observational studies, such as the *Nurse’s Health Study* which demonstrate beneficial and cardioprotective effects of estrogen [1, 2]. While MHT is effective for relieving menopausal symptoms, its impact on cardiovascular health and associated risks remain an area of dispute.

Many questions still surround the interaction of estrogens and their identified receptors (ERs) ERα, ERβ, and more recently the G protein-coupled estrogen receptor (GPER). The precise signaling actions of ERs and associated proteins are most likely dependent on the relative expression levels of all three ERs, or the ER expression profile, at the specific site of action. Tissue-specific and sexually dimorphic ER expression patterns may contribute to the differential effects of estrogen as well as the protective benefits observed in females but not in males. Moreover, ER profiles may aid the development of menopausal therapies that elicit only the desired effects.

Previously existing methodologies for transcript quantification, including quantitative real-time PCR (qPCR) and reverse transcription qPCR (RT-qPCR), estimate results based on an experimentally generated standard curve. This method limits accuracy when directly comparing data between discrete runs and tissue types, because standard curves are not identical across all reactions [3, 4]. Furthermore, levels of popular housekeeping genes including β-actin and GAPDH, which are used as internal controls in RT-qPCR, are not consistent among different tissues [5], leading to difficulty when comparing target gene levels in multiple tissues. In contrast, droplet digital PCR (ddPCR) is an established and validated method that allows direct comparison of multiple target sequences from discrete experiments due to absolute quantification [3, 6–8]. Utilizing ddPCR, the current study established the unique ER expression profile in 10 tissues from adult rats.

## Methods

### Tissue harvesting

All procedures were conducted in accordance with the National Institutes of Health Guide for the Care and Use of Laboratory Animals and approved and monitored by the Tulane Institutional Animal Care and Use Committee. Sprague-Dawley rats (six female, six male) arrived from Envigo at 12–14 weeks of age (RGD Cat# 737903, RRID:RGD_737903). After 1 week of acclimation, tissues were harvested and immediately immersed in 10 μl RNAlater (QIAGEN Cat# AM7020, RRID:SCR_008539) per 1 mg of tissue to preserve RNA integrity. Larger tissues were cut into slices less than 0.5 cm thick in accordance with the manufacturer’s instructions. Tissues were stored for 24 h at 4 °C before being archived at − 20 °C.

### Cell culture

Rat embryonic A7r5 aortic smooth muscle cells (ATCC Cat# CRL-1444, RRID:CVCL_0137) were cultured in 10 cm dishes with DMEM (Thermo Fisher Scientific Cat# 11330057, RRID:SCR_008452) containing 10% FBS (Sigma Cat# F6178, RRID:SCR_008988). After reaching approximately 80% confluency, media was replaced with DMEM containing 0.5% charcoal-stripped FBS (Sigma Cat# F6765) for 24 h to reduce to influence of hormones in the serum. Cells were washed, pelleted, resuspended in a small volume of PBS, then mixed with 5–10 volumes of RNAlater, and stored until analysis.

### RNA extraction

RNA extraction was accomplished using the RNeasy MiniKit (QIAGEN, Cat# 74106) according to the manufacturer’s protocol. Purity and concentration of RNA was determined by a NanoDrop™ 2000 Spectrophotometer (Thermo Fisher Scientific) using a previously described method [9]. Samples with a 260/280 ratio greater than 1.8 were used in the study.

### Droplet digital PCR

ddPCR was accomplished using a previously described method [6, 10]. Briefly, RNA was combined with One-Step RT-ddPCR Advanced Kit for Probes (Bio-Rad Laboratories, Cat# 1864021, RRID:SCR_008426) and the following PrimePCR Primers (all from Bio-Rad Laboratories): Gper, Rat (RefSeq: NM_133573, Fluorophore: FAM, Unique Assay ID: dRnoCPE5151056); Esr1, Rat (RefSeq: NM_012689, Fluorophore: HEX, Unique Assay ID: dRnoCPE5176827); Esr2, Rat (RefSeq: NM_012754, Fluorophore: FAM, Unique Assay ID: dRnoCPE5175914); and Cyp19a1, Rat (RefSeq: NM_017085, Fluorophore: HEX, Unique Assay ID: dRnoCPE5174813). Only two fluorescent signals were read for each sample (GPER-FAM/ESR1-HEX), and the same RNA sample was used to read the other two genes on a separate run (ESR2-FAM/CYP19A1-HEX). The reaction mixture was fractionated into more than 10,000 individual 1 nl droplets by oil emulsion microfluidics. Droplets were analyzed via the Bio-Rad QX200 droplet reader and QuantaSoft software and converted to copies/ng RNA based on the RNA concentration and the total volume added to the reaction. Samples were rerun or excluded if they had too many positive or negative droplets (does not satisfy Poisson statistics), Quantasoft Quality Scores below 0.85, or less than 10,000 droplets.

### Statistical analysis

GraphPad Prism (RRID:SCR_002798) was utilized for statistical analysis. Sex differences in transcript expression across tissues were analyzed using 2-way ANOVA with factors of sex and tissue followed by Sidak’s multiple comparisons test. ER mRNA expression profiles within each tissue were analyzed via two-way ANOVA with factors of gene and sex followed by Sidak’s multiple comparisons test. Cohen’s *d* was calculated as the difference between two means divided by the average standard deviation. For tissues found in only one sex (mammary gland and uterus) as well as for cell experiments, significant differences in gene expression were determined using one-way ANOVA and Sidak’s multiple comparison test.

## Results

**Fig. 1:**
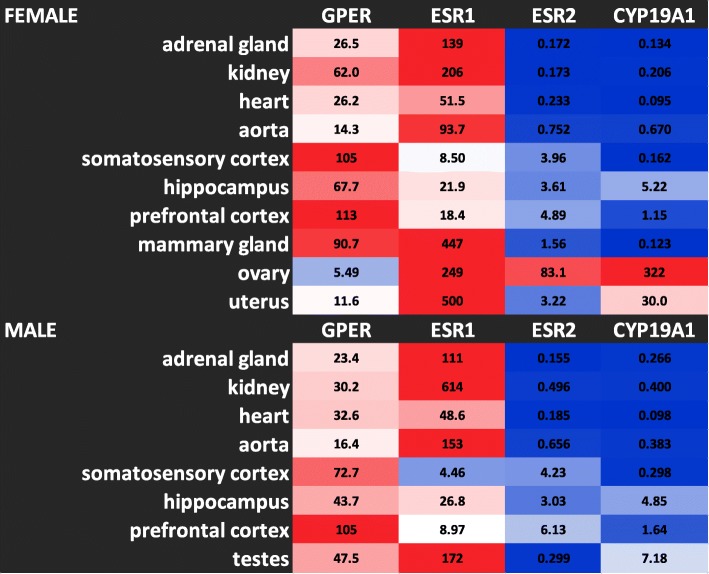
Heatmap of ddPCR results for GPER, ESR1, ESR2, and CYP19A1 in 10 tissue types from female and male Sprague-Dawley rats

An overview of all experimental data gathered from ddPCR analysis of GPER, ESR1, ESR2, and aromatase from the isolated total RNA of 10 cardiovascular, brain, and reproductive tissues is presented as a heatmap (Fig. 1).

**Fig. 2:**
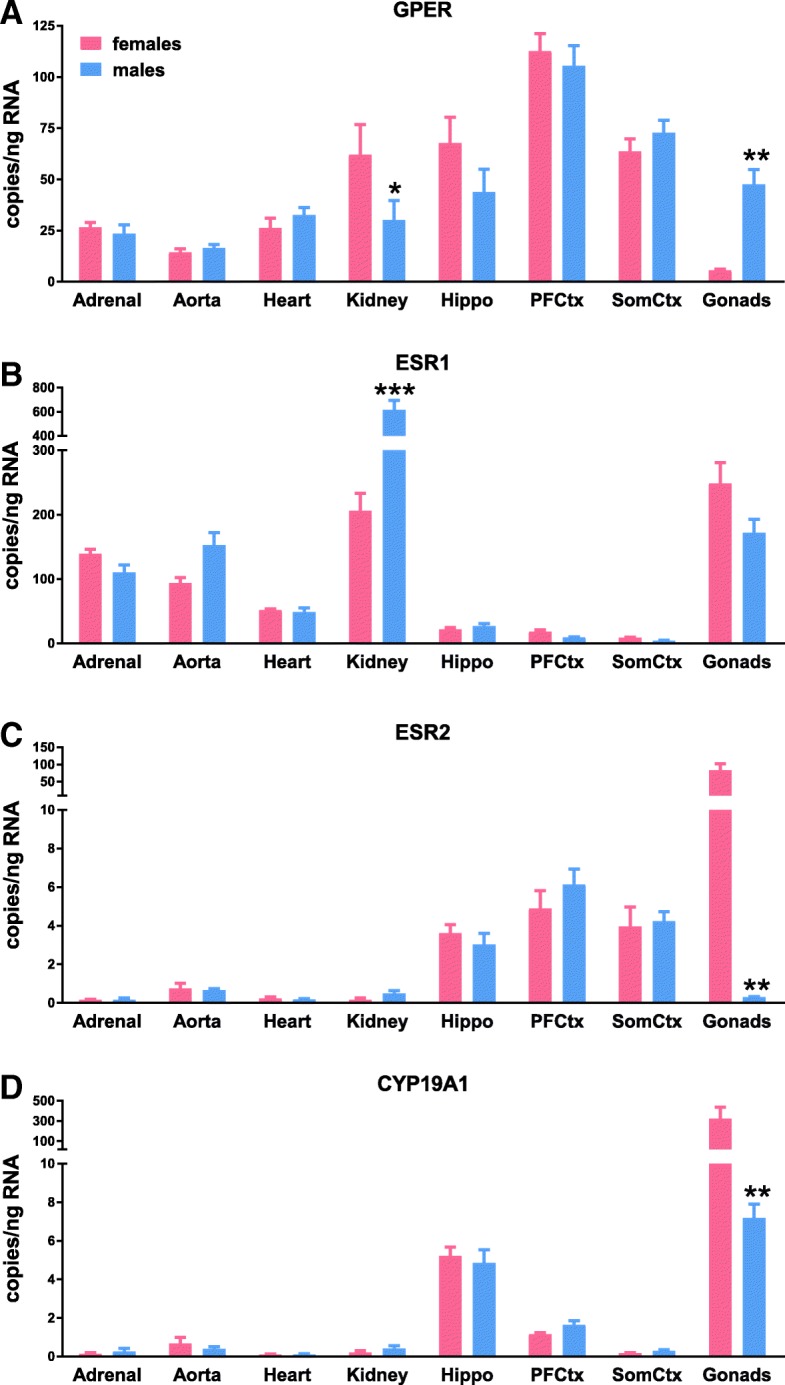
Expression of each gene was compared using two-way ANOVA (sex × tissue) with Sidak’s multiple comparisons test, *P < 0.05, **P < 0.01, and ***P < 0.001 vs. females. **a** GPER, **b** ESR1, **c** ESR2, and **d** CYP19A1

We next analyzed each gene individually in order to determine the effect of sex on estrogen receptor as well as the enzyme aromatase across all tissues (Fig. 2). Mammary and uterine samples were excluded since they could not be paired across sex. For GPER, there was no main effect of sex but tissue and the interaction were significant (Fig. 2a, Table 1). GPER was significantly lower in the ovary (5.5 ± 1.5 copies/ng RNA) and male kidneys (30 ± 23 copies/ng RNA) in comparison with tissues of the opposite sex (testes 48 ± 16 copies/ng RNA and female kidneys 62 ± 36 copies/ng RNA). A main effect was found for sex, tissue, and the interaction when analyzing expression of ESR1, ESR2, and CYP19A1 (Fig. 2b–2d, Table 1). Opposite of the sex difference in renal GPER, renal ESR1 was significantly greater in males. Also opposite to the predominance of GPER in the testes, ESR2, and CYP19A1 expression was significantly greater in ovaries.

**Table 1:** Statistical analysis of data presented in Fig. 2. Post hoc results are only shown for tests which reached statistical significance (*P* < 0.05)

	DF	eta squared Cohen’s *d*	F (DFn, DFd) t	*P* value
GPER (two-way)				
Interaction	7	8.56	F (7, 77) = 4.08	*P* = 0.0007
Tissue	7	67.1	F (7, 77) = 32.0	*P* < 0.0001
Sex	1	0.0137	F (1, 77) = 0.0459	*P* = 0.8310
>Kidney F vs. M	77	1.07	*t* = 2.96	*P* = 0.0327
>Gonads F vs. M	77	− 4.75	*t* = 3.72	*P* = 0.0030
ESR1 (two-way)				
Interaction	7	20.2	F (7, 79) = 19.2	*P* < 0.0001
Tissue	7	65.5	F (7, 79) = 62.3	*P* < 0.0001
Sex	1	1.91	F (1, 79) = 12.7	*P* = 0.0006
>Kidney F vs. M	79	− 3.08	*t* = 11.8	*P* < 0.0001
ESR2 (two-way)				
Interaction	7	37.4	F (7, 78) = 18.6	*P* < 0.0001
Tissue	7	34.8	F (7, 78) = 17.3	*P* < 0.0001
Sex	1	5.07	F (1, 78) = 17.6	*P* < 0.0001
>Gonads F vs. M	78	3.57	*t* = 12.2	*P* = 0.0030
CYP19A1 (two-way)				
Interaction	7	26.3	F (7, 78) = 7.06	*P* < 0.0001
Tissue	7	28.4	F (7, 78) = 7.62	*P* < 0.0001
Sex	1	3.67	F (1, 78) = 6.89	*P* = 0.0104
>Gonads F vs. M	78	2.19	*t* = 12.2	*P* = 0.0030

**Fig. 3:**
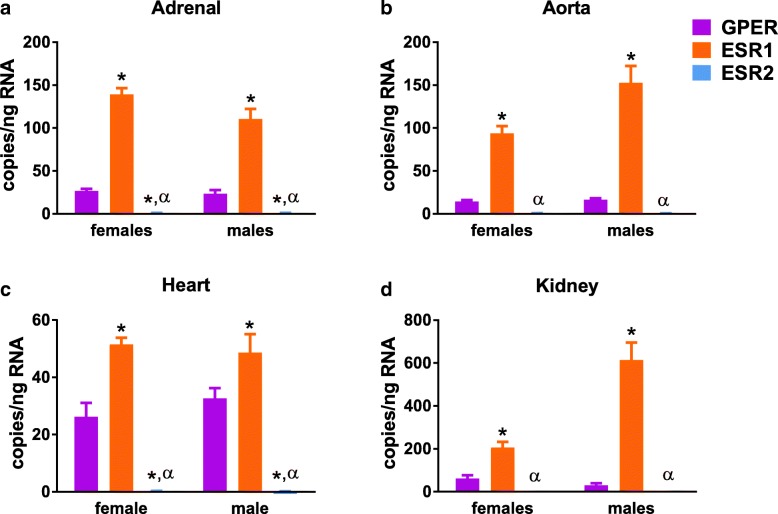
Estrogen receptor profiles in cardiovascular tissues. (**a**) Adrenal gland, (**b**) Aorta, (**c**) Heart, and (**d**) Kidney. Each tissue was compared using two-way ANOVA (sex × gene) with Sidak’s multiple comparisons test, *P < 0.05 vs. GPER, ^α^*P* < 0.05 vs. ESR1

Next, we determined the estrogen receptor profile for each tissue, grouped by function. Due to nearly undetectable levels of CYP19A1 except in reproductive tissues, this gene was not included in the analysis of cardiovascular or brain ER profiles. Cardiovascular tissues (adrenal, aorta, heart, and kidney) showed a predominance of ESR1, followed by GPER (Fig. 3 and Table 2). ESR2 had near undetectable levels in all cardiovascular tissues analyzed. In the adrenal gland and heart, but not the aorta or kidney, both male and female animals had significantly more ESR1 relative to GPER and ESR2. There was a significant effect of sex on gene expression in all tissues excerpt the heart. Higher levels of ESR1 were noted in the adrenal glands of females in comparison with males, but lower ESR1 was found in the female aorta and kidney.

**Table 2:** Statistical analysis of data presented in Fig. 3. Post hoc results are only shown for tests which reached statistical significance (*P* < 0.05)

	DF	eta squared Cohen’s *d*	F (DFn, DFd) t	*P* value
Adrenal (two-way)				
Interaction	2	1.309	F (2, 29) = 3.298	*P* = 0.0512
Sex	1	0.8823	F (1, 29) = 4.445	*P* = 0.0438
>ESR1: F vs. M	29	1.24	*t* = 3.347	*P* = 0.0068
gene	2	91.78	F (2, 29) = 231.2	*P* < 0.0001
>F: GPER vs. ESR1	29	− 9.530	*t* = 13.15	*P* < 0.0001
>F: GPER vs. ESR2	29	8.580	*t* = 3.079	*P* = 0.0135
>F: ESR1 vs. ESR2	29	15.81	*t* = 16.23	*P* < 0.0001
>M: GPER vs. ESR1	29	− 4.442	*t* = 10.17	*P* < 0.0001
>M: GPER vs. ESR2	29	4.313	*t* = 2.591	*P* = 0.0438
>M: ESR1 vs. ESR2	29	7.654	*t* = 12.29	*P* < 0.0001
Aorta (two-way)				
Interaction	2	5.10	F (2, 29) = 6.87	*P* = 0.0036
Sex	1	2.77	F (1, 29) = 7.47	*P* = 0.0106
>ESR1: F vs. M	29	− 4.60	*t* = 4.66	*P* = 0.0002
Gene	2	81.0	F (2, 29) = 109	*P* < 0.0001
>F: GPER vs. ESR1	29	− 2.72	*t* = 6.27	*P* < 0.0001
>F: ESR1 vs. ESR2	29	3.33	*t* = 7.34	*P* < 0.0001
>M: GPER vs. ESR1	29	− 7.31	*t* = 10.8	*P* < 0.0001
>M: ESR1 vs. ESR2	29	8.74	*t* = 11.4	*P* < 0.0001
Heart (two-way)				
Interaction	2	0.769	F (2, 30) = 0.804	*P* = 0.457
Sex	1	0.0665	F (1, 30) = 0.139	*P* = 0.712
Gene	2	84.8	F (2, 30) = 88.7	*P* < 0.0001
>F: GPER vs. ESR1	30	− 2.87	*t* = 4.76	*P* = 0.0001
>F: GPER vs. ESR2	30	4.29	*t* = 4.88	*P* < 0.0001
>F: ESR1 vs. ESR2	30	17.2	*t* = 9.64	*P* < 0.0001
>M: GPER vs. ESR1	30	− 1.29	*t* = 3.01	*P* = 0.0157
>M: GPER vs. ESR2	30	7.12	*t* = 6.09	*P* < 0.0001
>M: ESR1 vs. ESR2	30	6.05	*t* = 9.10	*P* < 0.0001
Kidney				
Interaction	2	18.6	F (2, 30) = 0.804	*P* < 0.0001
Sex	1	7.30	F (1, 30) = 0.139	*P* = 0.0002
>ESR1: F vs. M	30	− 3.08	*t* = 8.09	*P* < 0.0001
Gene	2	62.3	F (2, 30) = 88.7	*P* < 0.0001
>F: GPER vs. ESR1	30	− 2.81	*t* = 4.76	*P* = 0.023
>F: ESR1 vs. ESR2	30	6.18	*t* = 9.64	*P* = 0.0009
>M: GPER vs. ESR1	30	− 5.25	*t* = 3.01	*P* < 0.0001
>M: ESR1 vs. ESR2	30	6.16	*t* = 9.10	*P* < 0.0001

**Fig. 4:**
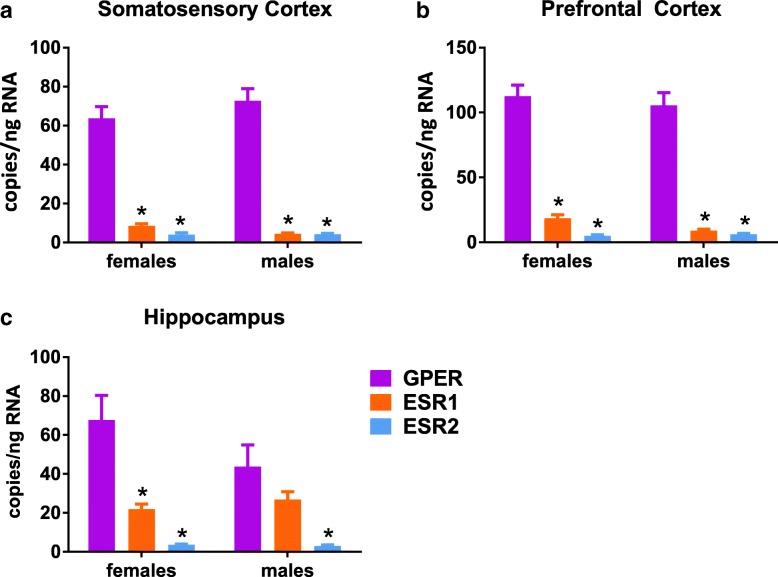
Estrogen receptor profiles in brain tissues. (**a**) Somatosensory Cortex, (**b**) Prefrontal Cortex, and (**c**) Hippocampus. Each tissue was compared using two-way ANOVA (sex × gene) with Sidak’s multiple comparisons test, *P < 0.05 vs. GPER

In contrast to data from cardiovascular tissues, GPER was the predominant transcript in the three selected brain regions (prefrontal cortex, somatosensory cortex, and hippocampus; Fig. 4 and Table 3). ESR1 and ESR2 displayed similarly low levels in the brain in both females and males. In addition, there was no main effect of sex in the brain. GPER was expressed significantly higher than ESR1 and ESR2 in female hippocampus, female and male prefrontal cortex, and female somatosensory cortex. In the male hippocampus and somatosensory cortex, GPER was significantly higher than only ESR2.

**Table 3:** Statistical analysis of data presented in Fig. 4. Post hoc results are only shown for tests which reached statistical significance (*P* < 0.05)

	DF	eta squared Cohen’s *d*	F (DFn, DFd) t	*P* value
Hippocampus (two-way)				
Interaction	2	5.06	F (2, 29) = 2.40	*P* = 0.109
Sex	1	1.44	F (1, 29) = 1.37	*P* = 0.252
Gene	2	60.2	F (2, 29) = 28.5	*P* < 0.0001
>F: GPER vs. ESR1	29	2.45	*t* = 4.77	*P* = 0.0001
>F: GPER vs. ESR2	29	4.00	*t* = 6.67	*P* < 0.0001
>M: GPER vs. ESR2	29	3.07	*t* = 4.03	*P* = 0.0011
Prefrontal cortex (two-way)				
Interaction	2	0.223	F (2, 30) = 0.517	*P* = 0.601
Sex	1	0.276	F (1, 30) = 1.28	*P* = 0.266
Gene	2	93.0	F (2, 30) = 216	*P* < 0.0001
>F: GPER vs. ESR1	30	6.75	*t* = 12.0	*P* < 0.0001
>F: GPER vs. ESR2	30	9.21	*t* = 13.8	*P* < 0.0001
>M: GPER vs. ESR1	30	7.05	*t* = 12.3	*P* < 0.0001
>M: GPER vs. ESR2	30	7.54	*t* = 12.7	*P* < 0.0001
Somatosensory cortex (two-way)				
Interaction	2	0.768	F (2, 29) = 1.84	*P* = 0.177
Sex	1	0.0811	F (1, 29) = 0.389	*P* = 0.538
Gene	2	91.4	F (2, 29) = 219	*P* < 0.0001
>F: GPER vs. ESR1	29	6.84	*t* = 4.767	*P* = 0.0001
>F: GPER vs. ESR2	29	7.52	*t* = 6.668	*P* < 0.0001
>M: GPER vs. ESR2	29	8.30	*t* = 4.031	*P* = 0.0011

**Fig. 5:**
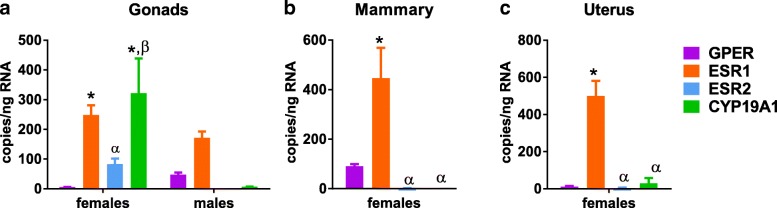
Estrogen receptor profiles in reproductive tissues. (**a**) Gonads, (**b**) Mammary gland, and (**c**) Uterus. Each tissue was compared using two-way ANOVA (sex × gene) with Sidak’s multiple comparisons test, *P < 0.05 vs. GPER, ^α^*P* < 0.05 vs. ESR1, ^ß^*P* < 0.05 vs. ESR2

The ER profiles of reproductive tissues (gonads, mammary gland, and uterus) were dominated by significantly greater ESR1 expression relative to GPER and ESR2 (Fig. 5 and Table 4). With the exception of the ovaries and uterus, CYP19A1 expression was nearly absent. In the ovary, CYP19A1 expression was equally as high as ESR1. Data from the testes did not reach the threshold of significance, though ESR1 mRNA tended to be higher than the three other surveyed genes.

**Table 4:** Statistical analysis of data presented in Fig. 5. Post hoc results are only shown for tests which reached statistical significance (*P* < 0.05)

	DF	eta squared Cohen’s *d*	F (DFn, DFd) t	*P* value
Gonads (two-way)				
Interaction	3	17.8	F (3, 38) = 5.33	*P* = 0.0037
Sex	1	12.2	F (1, 38) = 11.0	*P* = 0.0020
>CYP19A1: F vs. M	38	2.19	*t* = 4.95	*P* < 0.0001
gene	3	25.4	F (3, 38) = 7.62	*P* = 0.0004
>F: GPER vs. ESR1	38	− 6.01	*t* = 3.82	*P* = 0.0029
>F: GPER vs. CYP19A1	38	− 2.20	*t* = 4.97	*P* < 0.0001
>F: ESR2 vs. CYP19A1	38	− 1.44	*t* = 3.75	*P* = 0.0035
Mammary (one-way)				
Gene	3	64.5	F (3, 20) = 12.12	*P* < 0.0001
>F: GPER vs. ESR1	20	− 2.23	*t* = 4.13	*P* = 0.0031
>M: GPER vs. ESR1	20	2.98	*t* = 5.163	*P* = 0.0003
>M: GPER vs. ESR2	20	3.00	*t* = 5.18	*P* = 0.0003
Uterus (one-way)				
Gene	3	92.2	F (3, 8) = 31.7	*P* < 0.0001
>F: GPER vs. ESR1	8	− 6.66	*t* = 8.01	*P* = 0.0003
>M: GPER vs. ESR1	8	6.80	*t* = 8.14	*P* = 0.0002
>M: GPER vs. ESR2	8	4.94	*t* = 7.70	*P* = 0.0003

**Fig. 6:**
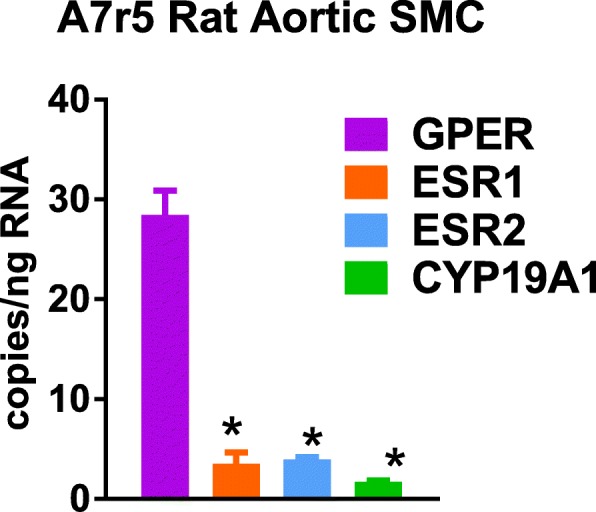
Estrogen receptor profile in rat embryonic aortic smooth muscle A7r5 cell line. One-way ANOVA with Sidak’s multiple comparisons test, *P < 0.05 vs. GPER

Since all of the samples tested thus far were whole tissue homogenates, we next determined the ER profile in A7r5 cells, a rat aortic smooth muscle cell line frequently used in our lab (Table 5). Since this cell line originated from rat embryos, we assumed that the sex of the cells was a combination of male and female. GPER expression was predominant in this cell type, with similarly low levels of ESR1, ESR2, and CYP19A1 (Fig. 6).

**Table 5:** Statistical analysis of data presented in Fig. 6. Post hoc results are only shown for tests which reached statistical significance (*P* < 0.05)

	*DF*	eta squared Cohen’s *d*	F (DFn, DFd) t	*P* value
Rat aortic SMC (one-way)				
Gene	3	88.1	F (3, 31) = 76.52	*P* < 0.0001
>F: GPER vs. ESR1	31	4.52	*t* = 12.09	*P* < 0.0001
>F: GPER vs. ESR2	31	5.90	*t* = 11.51	*P* < 0.0001
>F: GPER vs. CYP19A1	31	6.60	*t* = 12.57	*P* < 0.0001

**Fig. 7:**
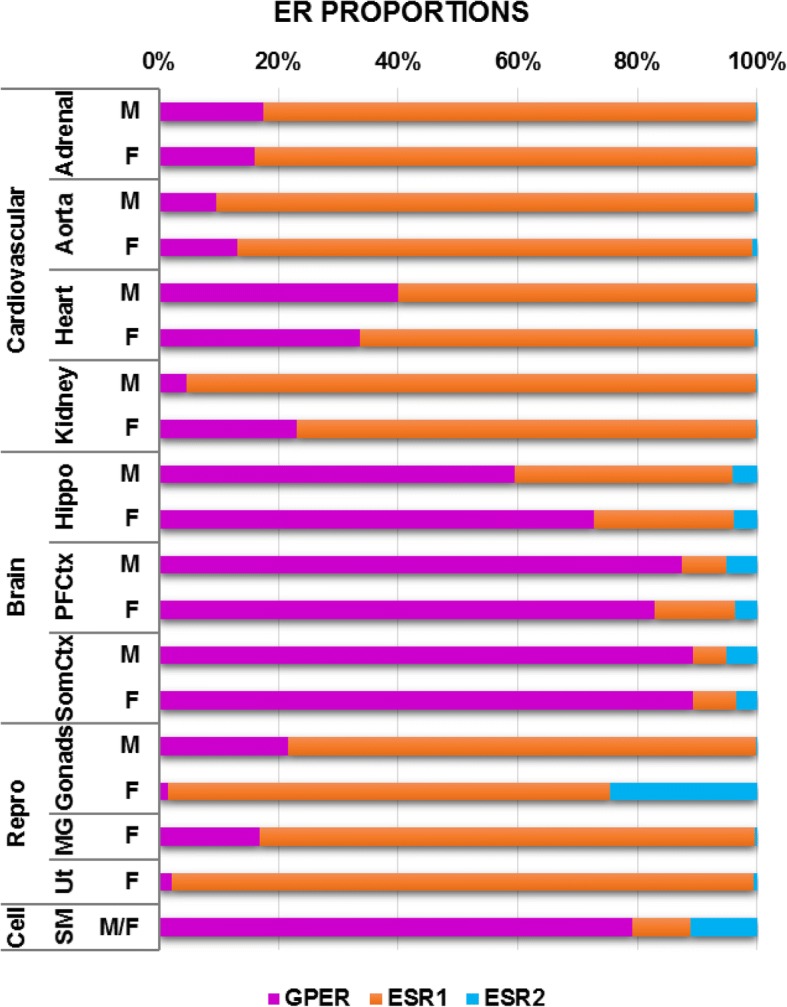
Estrogen receptor profiles for all tissues expressed as a percent of total receptor expression

In order to get a more thorough picture of the ER profile across tissues, Fig. 7 presents the normalized data for the three estrogen receptors across all samples.

## Discussion

The current study quantified and compared mRNA for the three known estrogen receptors and the enzyme aromatase in 10 tissues using a previously validated PCR technique that allows direct quantification of absolute transcript number. The data indicates a surprising lack of sexual dimorphism in key players for the primary female sex hormone. However, significant differences were noted in the range of receptor expression across tissues as well as the predominant estrogen receptor in each organ system. The data provide a guide for future studies by establishing the absolute amounts of estrogen receptor mRNA in a significant number of target organs.

Sex differences were broadly absent except in the kidneys (GPER and ESR1) and gonads (GPER, ESR2, and CYP19A1). The greater expression of renal ESR1 in male versus female Sprague-Dawley rats was also detected by others using the branched DNA signal amplification assay [11]. The direction of this sexual dimorphism is surprising considering that deletion of this gene in mice increases proteinuria and glomerular damage in females but not in males [12]. This same study found that genetic deletion of ESR2 does not impact renal health and reflects the nearly undetectable levels of this transcript in kidneys from both sexes in the current study. Similarly, kidneys from adult AKR mice lack ESR2 and have greater ESR1 in males compared with females [13]. Opposite of our ESR1 results, renal GPER was greater in females than males, which may compensate for lower ESR1 since its activation protects against renal damage in female rats [14]. The gonads showed the most sexual dimorphism, with greater ESR2 and CYP19A1 but less GPER in ovaries versus testes. Higher levels of ovarian ESR2 may underlie the more prominent reproductive phenotype in females with ESR2 deletion in comparison with males [15]. The lack of a sex difference in ESR1 between the testes and ovary was surprising considering the obvious differences in anatomy as well as sex hormones. Analysis by qPCR in zebrafish found that in contrast to our findings, ESR1 is significantly lower in the testes versus the ovaries [16]. However, their findings of greater CYP19A1 in female fish gonads supports our results and is most likely related to the more important role for this enzyme in sexual differentiation of females in comparison with males [17].

GPER was relatively stable across all tissues of both sexes, ranging from 5.5 copies/ng RNA (ovary) to 113 copies/ng RNA (prefrontal cortex), an approximately 20-fold difference. This finding is consistent with current literature that describes GPER expression as ubiquitous [18, 19]. In contrast, ERα and ERβ were variable across all tissues although relatively stable within each organ system. ESR1 displayed a range of 4.5 (somatosensory cortex) to 614 (kidney) copies/ng RNA, a fold change of ~ 136 while ESR2 ranged from 0.15 (adrenal gland) to 83 (ovary) copies/ng RNA or ~ 550-fold. The stability of GPER across all tissues of a given organ system as opposed to the more variable expression of ERα and ERβ may be indicative of the rapid signaling role of GPER [20–22]. In contrast, the transcriptional role of nuclear ERs may necessitate a more tailored pattern of receptor expression [23, 24]. GPER-mediated rapid signaling is demonstrated in tissues such as blood vessels, where it induces vasodilation via nitric oxide (NO) and cAMP signaling [20] and impacts calcium mobilization [25]. GPER activation also increases mitochondrial calcium retention in the heart within minutes [26] and decreases central control of food intake within an hour [27], both of which demonstrate the importance of this non-genomic pathway. The greater variability and tissue specificity for ESR1 and ESR2 may be important for regulating the large number of genes that are important for growth and reproduction. Genomic signaling by ERα and ERβ involves direct binding to estrogen-responsive elements, transcription factors, or cofactor complexes that regulate numerous genes [28]. This transcriptional role may necessitate a more tailored pattern of receptor expression [23, 24].

In cardiovascular tissues, ESR1 predominated over both ESR2 and GPER. The magnitude of ESR1 dominance was greatest in the kidney, where ESR1 was three times higher than GPER in females and sixfold greater in males. In the heart, ESR1 and GPER copy number was the most similar with a difference of 1.4-fold and 1.9-fold in males and females, respectively. Higher expression of GPER in the heart is consistent with findings of improved function as well as reduced remodeling in response to administration of the GPER agonist G-1 [29]. Studies using cardiomyocyte-specific deletion of GPER indicate that this cell type is likely the most important target for estrogenic actions mediated by GPER [30, 31]. ESR2 was nearly undetectable in the heart as well as all cardiovascular tissues, supporting other studies showing a lack of ESR2 in cardiac tissue and isolated cardiomyocytes from both sexes [32] as well as in the adult mouse kidney [13]. Since studies implicate this in attenuating the hypertrophic response to pressure overload in females [33], cardiac ESR2 expression may be induced in response to tissue injury or disease.

In the three brain areas analyzed, prefrontal cortex, somatosensory cortex, and hippocampus, GPER was dominant whereas ESR1 was about 75% lower and similar to levels of ESR2. Previous data generated in our laboratory reveals a similar expression pattern in the somatosensory cortex of Thy1 female mice, where sensory-evoked structural plasticity positively correlates with high estrogen stages of the estrus cycle [34]. These results suggest a conserved role for rapid estrogen signaling via GPER in telencephalic brain regions. In addition, GPER reduces inflammatory markers in primary cultures of microglial cells when treated with the selective agonist G1, indicating a neuroprotective role for this receptor [35]. Although expression of ESR2 was much lower than the other receptors throughout the study, the amount found in brain was greater relative to all other tissues except the ovaries, suggesting an important role in nervous tissue. In this regard, ESR2, but not ESR1, has been recently found responsible for the modulation and promotion of synaptogenesis in cortical neurons in vitro [36] and in vivo [37], indicating a crucial role of this receptor in brain function, especially for memory and cognition. Furthermore, the relative ESR2/ESR1 expression ratio is increasingly appreciated as important in memory performance and cognitive decline with respect to age [38].

ESR1 was the dominant gene in gonadal tissues of both sexes, which is in accordance with data establishing it as a vital receptor in animal fertility [39]. Second to ESR1, males had significant levels of GPER but low ESR2, while females exhibited the inverse relationship. Higher levels of ESR2 versus GPER in the ovary and uterus supports the presence of smaller litters in ESR2 knockout animals [15, 39] and the lack of any reproductive changes in GPER knockout mice [40, 41] or rats treated with the GPER agonist [42]. Opposite to females, genetic deletion of ESR2 in males does not alter fertility [39] and correlates with our data showing low receptor levels in the testes. Despite the higher expression of GPER in males, genetic deletion of this receptor is reported to not alter fertile or function of the hypothalamic-pituitary-gonadal axis [40, 41]. Since the ovaries are the primary source of circulating estrogen, it was not surprising that CYP19A1 expression was detected at very high levels in this tissue. The low levels in most other tissues (except testes and hippocampus) are consistent with previous reports of extragonadal CYP19A1 expression only in the brain and adipose tissue, which are also major sites for estrogen production [43].

The most commonly used molecular assay in biological sciences is currently qPCR [44]. This technique utilizes nucleic acid primers for a gene of interest, a fluorescent reporter dye, putative formulae, or a standard curve of known nucleic acid concentrations in order to approximate the concentration of transcript by comparing fluorescence intensity over time [45]. A baseline value of background fluorescence is established, and by determining the cycle number at which fluorescence surpasses background levels one can establish the starting concentration of the target sequence. ddPCR was developed to more accurately and reproducibly quantify nucleic acids without the use of standard curves or reference genes [3]. Unlike qPCR, ddPCR accomplishes absolute quantification by partitioning the starting materials into thousands of nanoliter droplets suspended in an oil emulsion containing one or zero target sequences. After amplification by traditional PCR, droplets are quantified as positive or negative, and the starting concentration of target can be calculated using Poisson statistics. Because ddPCR is based on analysis of a binary end state, it is far more resistant to contamination and fluctuations in PCR efficiency, both of which drastically affect results obtained via qPCR [4]. A comparison of RT-qPCR and ddPCR in quantifying strains of influenza found that ddPCR was 30-fold more sensitive than RT-qPCR and results obtained by ddPCR were more precise [8], supporting conclusions in other investigations [3, 46]. Overall, several studies find ddPCR to be more reliable, reproducible, sensitive, and consistent when compared to traditional qPCR methods with the added benefit of not requiring standard curves or normalization to a reference gene [3, 4, 8, 47].

One limitation of utilizing tissue homogenates is a heterogeneous mixture of differentiated cell types. Analysis of mRNA from the rat aortic smooth muscle cell A7r5 cell line resulted in a completely different ER profile than that obtained using the whole rat aorta, supporting the idea that each cell or subpopulation of cells may have an individual expression profile. The cell versus tissue data shows a similar paucity of ESR2, but GPER rather than ESR1 predominates implying variable responses to estrogens within the tissue and a need for cell-specific expression profiles. Other studies also show that gene expression profiles show low correlation with cell lines [48], indicating that different environments may have a strong influence on gene expression within the same tissue type.

Additional limitations are that protein levels may not correlate with mRNA levels or patterns, and gene expression does not take into account the presence of multiple splice variants for estrogen receptors [49]. In fact, Irsik et al. found that ERß protein is expressed at similar levels in the mouse kidney and ovary, while we found that ERß mRNA was more than 400 times lower in the kidney versus ovary. These disparate findings suggest that either translation to protein does not follow the patterns seen here for mRNA or that there is a lack of specificity of currently available antibodies. Indeed, care needs to be taken to validate all antibodies against a control sample where the protein of interest has been deleted [50]. Nevertheless, the method used here is to our knowledge the best method for quantification and comparison of four genes of interest in different tissues.

## Conclusions

In conclusion, this study revealed a wide range of ER expression across tissues, with surprisingly few sex differences. As illustrated in Fig. 7, which represents the data as parts of a whole, ER profiles were relatively consistent across tissues of the same organ system, with large differences in the ER profile between systems. The relative stability of GPER mRNA across all tissues as opposed to the variable expression of ESR1 and ESR2 is perhaps best explained by the differing role that GPER plays in relation to the nuclear ERs. Consistent expression of GPER allows all tissues to respond rapidly and concertedly to estrogen signaling, while the differential expression of ESR1 and ESR2 allows for tissue-specific transcriptional regulation in response to the same signal. The predominance of GPER in brain and ESR1 in cardiovascular tissues, and moreover the relative ratio between these receptors may serve to further tailor the response to estrogen in different tissues. Future studies will examine the impact of aging and disease on tissue-specific ER profiles. Overall, this work is indicative of the complex nature of ER research and the nuances that remain to be elucidated. Moreover, this study provides a contextual framework to guide future work on ER roles in specific organs.

## Abbreviations

AMP: Adenosine monophosphate
CEE: Conjugated equine estrogen
CVD: Cardiovascular disease
ddPCR: Droplet digital polymerase chain reaction
ER: Estrogen receptor(s)
Estradiol: 17β-estradiol
GMC: Glomerular mesenchymal cell(s)
GPER: G protein-coupled estrogen receptor
MHT: Menopausal hormone therapy
MPA: Medroxyprogesterone acetate
NHS: Nurse’s Health Study
PCR: Polymerase chain reaction
qPCR: Quantitative polymerase chain reaction
RT-qPCR: Reverse transcription quantitative polymerase chain reaction
VSMC: Vascular smooth muscle cell(s)
WHI: Women’s Health Initiative
